# Novel recombinant keratin degrading subtilisin like serine alkaline protease from *Bacillus cereus* isolated from marine hydrothermal vent crabs

**DOI:** 10.1038/s41598-021-90375-4

**Published:** 2021-06-07

**Authors:** Revathi Gurunathan, Bin Huang, Vinoth Kumar Ponnusamy, Jiang-Shiou Hwang, Hans-Uwe Dahms

**Affiliations:** 1 Department of Medicinal and Applied Chemistry, Kaohsiung Medical University, Kaohsiung City, 807 Taiwan; 2 Department of Biomedical Science and Environmental Biology, Kaohsiung Medical University, Kaohsiung City, 807 Taiwan; 3 Regenerative Medicine and Cell Therapy Research Center, Kaohsiung Medical University, Kaohsiung City, 80708 Taiwan; 4 Institute of Marine Biology, National Taiwan Ocean University, Keelung, 20224 Taiwan; 5 Center of Excellence for Ocean Engineering, National Taiwan Ocean University, Keelung, 20224 Taiwan; 6 Center of Excellence for the Oceans, National Taiwan Ocean University, Keelung, 20224 Taiwan; 7 Research Center for Environmental Medicine, Kaohsiung Medical University, Kaohsiung City, 807 Taiwan; 8 Department of Marine Biotechnology and Resources, National Sun Yat-Sen University, Kaohsiung City, 804 Taiwan

**Keywords:** Biochemistry, Biotechnology, Chemical biology

## Abstract

Microbial secondary metabolites from extreme environments like hydrothermal vents are a promising source for industrial applications. In our study the protease gene from *Bacillus cereus* obtained from shallow marine hydrothermal vents in the East China Sea was cloned, expressed and purified. The protein sequence of 38 kDa protease SLSP-k was retrieved from mass spectrometry and identified as a subtilisin serine proteinase. The novel SLSP-k is a monomeric protein with 38 amino acid signal peptides being active over wide pH (7–11) and temperature (40–80 °C) ranges, with maximal hydrolytic activities at pH 10 and at 50 °C temperature. The hydrolytic activity is stimulated by Ca^2+^, Co^2+^, Mn^2+^, and DTT. It is inhibited by Fe^2+^, Cd^2+^, Cu^2+^, EDTA, and PMSF. The SLSP-k is stable in anionic, non-anionic detergents, and solvents. The ability to degrade keratin in chicken feather and hair indicates that this enzyme is suitable for the degradation of poultry waste without the loss of nutritionally essential amino acids which otherwise are lost in hydrothermal processing. Therefore, the proteinase is efficient in environmental friendly bioconversion of animal waste into fertilizers or value added products such as secondary animal feedstuffs.

## Introduction

Shallow-water hydrothermal vents are unique environments, mostly with toxic ambience for animals, plants, and several microorganisms. Such hydrothermal vents occur near coastal volcanic regions^1^. The crab *Xenograpsus testudinatus* lives in shallow-water hydrothermal vents which are rich in sulfur, have highly fluctuating pH, and elevated temperatures, near Kueishantao (also called Turtle Island), NE-Taiwan^2^. This crab is endemic to this vent field and considered as one of the few known HV species found at depths < 200 m^1,3^. Marine-derived products and the genes of organisms found in extreme conditions like hydrothermal vents at varying temperature, pressure, and heavy metal concentrations are gaining interests in applied marine biotechnological research. Enzymes like proteases that can hydrolyse peptide bonds of proteins are valued in the drug designing industry, as well as for the production of detergents, and for environmental waste water treatment. The Enzyme Commission classifies proteases into six families: serine protease (EC 3.4.21), cysteine (EC 3.4.22), aspartic protease (EC 3.4.23), serine carboxy protease (EC 3.4.16), metalloprotease I (EC 3.4.24) and metallo-carboxy-protease (EC 3.4.17)^4^. Alkaline proteases can have a serine centre or can be of the metallo-type with optimal activity at neutral to alkaline pH. Thermostable bacterial proteases which can withstand alkaline conditions can be cloned and produced in large amounts^5–7^. Alkaline proteases received increasing attention in the 1960s when they were used as detergents in the detergent industry. Most of such detergents were produced by *Bacillus* spp. which subsequently provided about 35% of the microbial protease enzymes sold worldwide^5–7^. *Bacillus* sp. such as *Bacillus* sp. SSR1^8^, *Bacillus brevis*^9^, and *Bacillus stearothermophilus*^10^, produce alkaline proteases with a potential for detergent production. Protease activity depends highly on pH, ionic strength, temperature, and mechanical handling. Enzymes with novel properties and the ability to withstand harsh chemical treatments are of particular industrial demand.

Keratinase degrades keratin which in turn is a protective protein. It is highly rigid, recalcitrant and cannot be hydrolysed by other proteases. Keratinases, based on their active site are classified as serine proteases, serine metalloproteases, or metalloproteases^11^. Some keratinases belong to serine proteases (S8 family) and the superfamily of subtilisin-like proteases with an active serine centre^12^. Keratinases (EC 3.4.21) can withstand wide ranges of pH and temperature and show the ability to break down highly complex proteinaceaous structures like feathers, silk, collagen, horn, wool, hair, elastin, azokeratin, nails and the stratum corneum of eyes^11^. Keratinases degrade feathers which are otherwise considered as biological waste that is difficult to degrade and recycle^13^. The conventional chemical method of keratin degradation is using lime-sulfide. A drawback of this process is that large sulfide amounts are produced which are toxic, having a high biological oxygen demand (BOD) and chemical oxygen demand (COD), and producing a high amount of total suspended solids (TSS)^14^. To overcome the risk associated with the degradation of keratin substrates, the microbial keratinases are studied extensively in bio-cycling of agro-industrial wastes in cost-effective and eco-friendly ways^15^. Keratinase based formulations like Valkerase, Versazyme, CIBENZA, DP100 and FEED-0001 are useful for the improvement of the nutritional values of animal feeds^16^.

In this study we characterized a novel extracellular protease SLSP-k from bacteria associated with hydrothermal vent crabs. The objectives of this study were: (1) to amplify the gene for the novel SLSP-k, (2) to purify and characterize this protease enzyme, and (3) to explore the applications of the protease in research, value-added product synthesis, and biological waste treatment.

## Materials and methods

### Isolation and screening of microorganisms

Sampling was done at the hydrothermal vent site at Kueishantao (also called Turtle Island), an island in the East China Sea, part of Toucheng Township, Yilan County, Taiwan. Kueishantao is situated 9.1 km east of Kengfang Fishery Harbor^3^. We focused particularly on the isolation of bacteria from the vent crab *Xenograpsus testudinatus* at Kueishantao. All the bacterial strains isolated from this vent crab were screened for protease production using agar plates based on skim milk by measuring the zone of hydrolysis. Based on the highest proteolytic zone produced on skim milk agar plates, a bacterial strain was selected that was sequenced using the bacterial barcoding gene, 16S rRNA gene, applying the universal primers 27F and 1492R^17^. The sequence was edited by chromas 2.2 software and BlastN sequencing was performed followed by the construction of a phylogenetic tree using MEGA-X software^18^.

### Amplification of the serine protease gene

Primers used for the polymerase chain reaction were forward primer 5′-CGGGATCCCACRAATACTTCAAGYGCTGA-3′ and reverse: 5′-CGGAATTCGCATTGACTCTACCRTTTTTCCA-3′^19^. The genomic DNA was isolated using a genomic DNA isolation kit according to the instructions of the company (NucleoSpin, Microbial DNA, MACHEREY-NAGEL, Dȕren, Germany). PCR was performed with 2 μL of DNA extracted as a template (50 ng), 2.5 μM of each primer, 10× PCR buffer and 2 U of Taq polymerase (Invitrogen), 0.5 mM dNTPs in a 25 µL reaction. Polymerase reaction (T100 Thermal cycler, Bio-Rad, Hercules, California, USA) with initial denaturation at 95 °C for 5 min, repeated 34 cycles of denaturation at 95 °C for 1 min, annealing at 58 °C for 1 min, extension at 72 °C for 1 min and a final extension at 72 °C for 10 min was done. The amplified gene was eluted by a Gel purification kit (Mini Plus Plasmid DNA extraction System, Viogene, Taipei, Taiwan). The gel eluted product was sent out for sequencing, and bacterial identification was confirmed using BLAST at NCBI. The PCR product was used for cloning.

### Transformation in E. coli host cells

The PCR product was cloned in T and A cloning vectors. The ratio of vectors to insert was 1:3. The ligation reaction was set up according to the manufacturers protocol. In brief, 10 µL of reaction volume with 10 × diluted ligation buffer, 1 µL T4 DNA ligase insert to vector ratio of 3:1, respectively, was added and kept at 4 °C overnight. The ligation was confirmed by agarose gel electrophoresis. Transformation was carried out in one shot of *E. coli* (ECOS 101 DH5α) competent cells according to the manufacturers protocol. Briefly, the cells were thawed and 2.5 µL of ligation mixture were mixed and vortexed. The mix was incubated on ice and a temperature shock at 42 °C was provided for 40 s. The cells were then plated on pre-warmed plates with LB agar (0.5 mM IPTG, 40 µg/mL ampicillin, and 40 µg X-gal) and incubated overnight. Positive white colonies were selected and confirmed by colony PCR and plasmid sequencing.

Digestion of plasmid vector T and cloning vector A was done by HIND-III restriction enzymes in the presence of NEB buffer. The reaction was set up with vectors having 1000 ng concentration, enzyme 5 U, and buffer and were incubated overnight at 37 °C. Restriction was confirmed by agarose gel electrophoresis and HIND-III used for ligation in the expression vector. The expression vector pET-32b (+) was also digested using HINDIII restriction enzyme and confirmed by agarose gel electrophoresis. The cut vector pET32b+ and Insert was ligated using T4 DNA ligase enzyme at a ratio of 1:3, respectively, and incubated overnight at 4 °C. The transformation was performed in one shot ECOS BL21 (DE3) *E. coli* cells, following the manufacturer’s instructions. Briefly, 3.5 µL of the ligated product was mixed with competent cells [*E. coli* BL21 (DE3)] and kept on ice for 5 min right after a heat shock of 42 °C was provided for 40 s and plated on pre-prepared warmed plates with amp x-gal and IPTG. Blue white screening was used to identify positive colonies, colony PCR, and were finally confirmed by the Sanger sequencing method^20^. The BLASTN database of NCBI was used for sequence similarity search. Homology alignment was done with the Clustal Omega program. By selecting the sequence with the highest similarity, phylogenetic tree was constructed using Mega-X software^18^.

### Optimization of induction conditions for the expression of SLSP-k in E. coli (DE3)

Transformed *E. coli* BL21 (DE3) cells were grown in 10 mL of LB medium with 50 μg/mL of ampicillin at 37 °C by shaking overnight. The primary culture was inoculated into four 50 mL tubes at a ratio of 1:10. To determine the optimum induction temperature, recombinant *E. coli* BL21 (DE3) were grown at 37 °C until the absorbance 0.6 was reached at OD_600_. The bacterial culture was induced with 0.5 mM IPTG and 1 mM IPTG and incubated at 37 °C and 27 °C at each temperature for up to 10 h. One mL sample was taken every 1 h from T3 to T10 at 37 °C and 27 °C. The cell pellets were suspended in phosphate buffer and sonicated for 5 min with 20 s pulses. The samples were centrifuged at 13,000 rpm for 10 min and the supernatants were analyzed by SDS-PAGE.

### Lysis buffer selection

Five different lysis buffers, listed in Table 1, were used to lyse the cell pellets. The supernatant was analyzed by SDS PAGE. The buffer with highest yield of soluble recombinant protein was selected for further studies. From each buffer 2 mL were added to the cell pellet from a 5 mL IPTG-induced culture and sonicated for 5 min duration. The resulting lysed sample was centrifuged at 13,000 rpm for 10 min and the supernatant was then purified.

**Table 1:** Buffers and their composition used in the optimization of expression of recombinant SLSP-k.

S. no.	Buffer	Final concentration
1	Tris–HCl (Merck), pH 7.5	20 mM
	Dithiothreitol (DTT) (Sigma-Aldrich)	0.1 mM
	Lysozyme (Sigma-Aldrich)	1 mg/mL
2	Tris–HCl (Merck), pH 7.5	20 mM
	NaCl	0.5 mM
	Lysozyme (Sigma-Aldrich)	1 mg/mL
3	Phosphate buffer	20 mM
	NaCl	0.5 mM
	Urea	8 M
	Triton-X 100	1%
4	Phosphate buffer	20 mM
	NaCl	0.5 mM
	Triton-X 100	1%
5	Phosphate buffer	20 mM
	Triton-X 100	0.5 mM

### Purification of recombinant SLSP-k enzyme

The supernatant was filtered on a 0.22 µm filter and eluted by Ni Sepharose 6 fast flow resin in PD10 columns to elute the binded his-tagged proteins. Binding buffer with 20 mM imidazole eluted the binded his-tagged proteins and unbound proteins were washed using the washing buffer. Elution buffer at two different concentrations, 200 mM and 500 mM, was added to elute the his-tagged proteins to check for highest soluble recombinant proteins.

### Zymography and SDS-PAGE

The molecular weight of the soluble recombinant purified SLSP-k protein was studied by SDS-PAGE with stacking gel (4%) and resolving gel (12%). Zymography to check protease activity using 10 mg/mL gelatin was performed. The zymography gel electrophoresis was run at 100 V and 4 °C (BIO-RAD, Hercules, California, USA). The gel was washed in Triton X-100 (2.5%) solution at 37 °C for 30 min at gentle shaking. The gel was kept overnight in the developing buffer (pH 7.5) comprising of Tris base, CaCl_2_, ZnCl_2_, NaCl, and Brij 35 at 37 °C. Coomassie brilliant blue R-250 (0.1%) was used for 1 h each staining and de-staining (water: methanol: glacial acetic acid at ratios of 5:4:1) until clear bands visibly appeared, indicating protease activity on the gel.

### Mass spectrometry analysis of the purified protein

The band of the SDS gel was excised and destained. Trypsin digestion was performed at 37 °C for 4 h (In-Gel Tryptic Digestion Kit, Thermo Fisher Scientific) in order to identify the peptide sequence by mass spectrometry (MS). Desalting of the tryptic digested peptides were performed on a C18 proteomic column (Mass Solution Ltd., Taipei, Taiwan). MS analysis of the resulting peptides applying nLC/Q-TOF (Micromass, Manchester, UK) was performed. The resulting MS data were used to search against entries in the NCBI database using the MASCOT search program (Matrixscience, London, UK). Additionally, peptides with acetylated lysines were predicted. The parameters searched for were: mass values: monoisotopic; fragment mass tolerance: ± 0.4 Da; protein mass: unrestricted; maximal missed cleavages: 1; peptide mass tolerance: ± 0.4 Da; variable modification: oxidation in methionine; acetylation in lysine: carbamidomethylation in cysteine.

### Bioinformatic analysis

Protein sequence similarity and phylogenetic analysis was done applying the blastp program at NCBI, https://blast.ncbi.nlm.nih.gov/Blast.cgi. The sequences were selected on the basis of similarity percentage identity. For multiple sequence alignment we used the Clustal Omega program (https://www.ebi.ac.uk/Tools/msa/clustalo/). The I-TASSER structure prediction program was used to predict structures which used COFACTOR and COACH tools. COFACTOR can retrieve ligand-binding sites, EC and GO, by comparing the already available structures. Meta-server COACH provides output by combining data from multiple functional annotations (from the COFACTOR, S-SITE, and TM-SITE)^21^ (https://zhanglab.ccmb.med.umich.edu/I-TASSER/). To determine the signal peptide region SignalP server was used (http://www.cbs.dtu.dk/services/SignalIP/). A Phylogenetic tree was constructed by MEGA-X software. The confidence of the branching value was tested by bootstrapping 500 iterations. The final structures were retrieved from Discovery studio program for high quality images^22^.

### FT-IR analysis of casein hydrolysates

The hydrolysis of casein by SLSP-k was measured by highly sensitive FT-IR techniques. Enzyme and casein was mixed at equal volumes at optimal conditions, i.e. at 50 °C and pH 10.0 kept for 30 min. The hydrolysed product was centrifuged at 10,000 rpm for 10 min at 4 °C and the supernatant was collected. The obtained supernatant was freeze-dried overnight. FT-IR spectroscopy was performed by mixing 225 mg dried KBr (10% w/w) with 25 mg freeze dried hydrolysate^23^.

### Biochemical characterization

#### Protease activity assay

Proteolytic activity was assayed with casein (0.6%) as a substrate by Folin–Ciocalteu method with slight modifications^24^. The reaction was carried out with 1 mL of enzyme and 1 mL of substrate at 37 °C for 30 min. The reaction was stopped by adding 1 mL of 10% TCA (trichloroacetic acid), incubated at room temperature for 15–20 min and centrifuged at 5000 rpm for 10 min. Spectrophotometric absorbance reading was taken after mixing 1.0 mL of supernatant with 650 µL of 0.5 M Na_2_CO_3_ and 500 µL of two times diluted Folin–Ciocalteu reagent. The absorbance reading was taken by a UV spectrophotometer after 30 min of incubation at 660 nm against the blank sample.

#### Determination of optimum protease conditions

Enzyme activity was observed at varying temperatures ranging from 40 to 100 °C. For this purpose, 500 μL of the 0.6% (w/v) casein solution was mixed with 500 μL of enzyme solution followed by incubation for 1 h. Activity was studied according to a standard assay at each temperature. The relative activity was measured by keeping the highest activity as 100%. Thermal stability was determined with 3500 μL of the enzyme solution being kept in a water bath at a temperature ranging from 40 to 100 °C for 7 h. From the total mixture, a volume of 500 µL of enzyme was taken for reading after every 1 h. The relative activity (%) was calculated from the absorbance value.

SLSP-k activity was measured at varying pH values ranging from highly acidic to alkaline (2–12 pH). Since the protein was eluted and showed maximum solubility in phosphate buffer, the same buffer was used to predict the optimal pH for hydrolytic activity. Diluted enzyme solution in respective buffer (500 μL) was mixed with 0.6% casein solution in a total reaction volume of 3 mL followed by 1 h water bath at 50 °C incubation. The highest absorbance value was accepted as 100% and the relative activity from the absorbance (%) was predicted.

To find the effect on SLSP-k activity with the treatment of metal ions, such as monovalent metal ions (Na^+^ and K^+^), divalent metal ions (Ca^2+^, Co^2+^, Cu^2+^, Cd^2+^, Mn^2+^, Pb^2+^, Hg^2+^, Ni^2+^), and trivalent Fe^3+^ were used. Metal solutions (500 µL) at concentrations of 1 mM, 5 mM, and 500 μL of enzyme solution were mixed followed by incubation for 1 h at 50 °C. The residual hydrolytic activity was predicted from the absorbance^23,25^.

To study the effect of inhibitors PMSF, EDTA, and DTT were used at final concentrations of 1 mM and 5 mM. In this study 500 μL of inhibitor solution was stirred with 500 μL of enzyme solution and incubated for 30 min. Then the standard protease activity assay was performed and residual activity was calculated.

To study the stability of surfactants, 1 mM and 5 mM of SDS, Tween-20, Triton-X 100 was used. The surfactant solution (500 μL) was added to 500 μL of enzyme solution and incubated for 1 h and later a standard protease assay was performed as mentioned in “Protease activity assay”. The residual activity of SLSP-k was calculated.

The stability of SLSP-k was analysed after treatment with solvents like DMSO, ethanol, ethyl acetate, methanol, 2-propanol, acetone, acetonitrile, and NaCl. In this treatment, 100 μL of organic solvent were added to 900 μL of enzyme solution, kept for 1 h at 50 °C. A sample without the treatment of any organic solvent was kept as a control. Finally, the residual activity (%) of the enzyme was calculated from the absorbance value.

The activity of the SLSP-k for kinetic studies to calculate the V_max_ and Km with varying final concentrations of casein as substrate was studied from 2 to 20 mg/mL in phosphate buffer at pH 10 at 50 °C. The maximum velocity V_max_ and the Michaelis–Menten constant Km was calculated from Lineweaver–Burk plots^26^**.**

To check for keratinase activity, chicken feathers and human hair were treated with 500 µL of SLSP-k in phosphate buffer and incubated at 50 °C for 48 h. The samples were dried completely to remove excess water at 60 °C and using SEM analysis by drying and fixing the samples on carbon tape, and sputtering them with gold^27^.

Since human hair was used in the study, our ethical compliance statements are required and stated as following: (a) All methods were carried out in accordance with relevant guidelines and regulations; (b) We confirm that all experimental protocols were according the institutional regulations, namely that there was no formal permit or license required for hair harvested from the first author’s GR hairbrush for the above experiment; (c) No written informed consent was needed since the only subject (GR—see above) providing human hair from her hairbrush was over 18 years of age—and no parent and legal guardian was required—when obtaining hair without pain from her hairbrush.

## Results and discussion

### Protease activity and gene amplification

**Figure 1:**
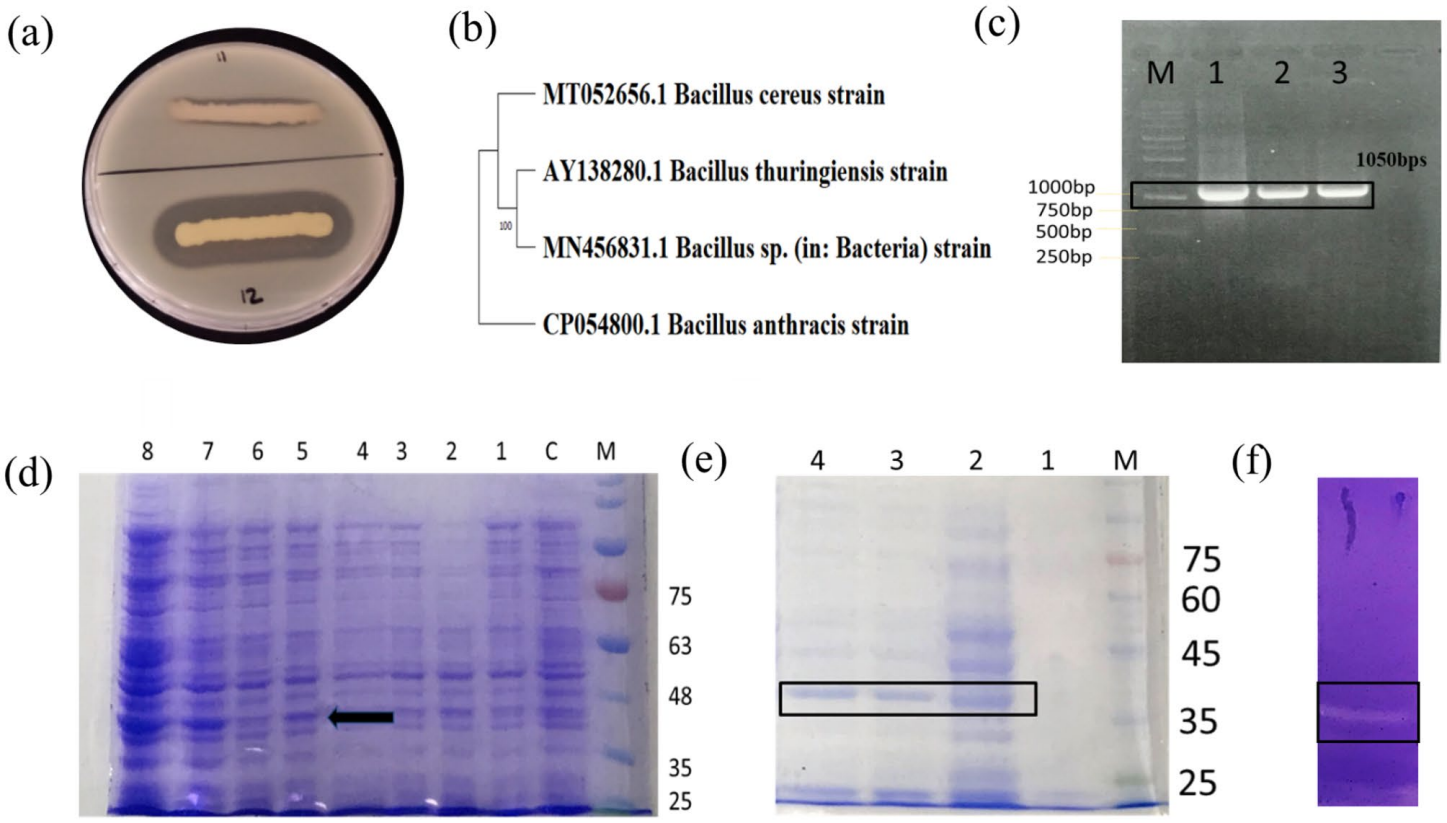
Protease assay and phylogenetic tree. (**a**) Protease activity assay by Skim milk assay agar of *Bacillus cereus*, (**b**) The evolutionary history was inferred by using the Maximum Likelihood method using MEGA X software. The tree with the highest log likelihood (− 160,829.40) is shown. The percentage of trees in which the associated taxa clustered together is shown next to the branches, (**c**) The PCR amplified gene (1050 bps gene amplified by specific primers), (**d**) The SDS-PAGE analysis of protein expression. Lane M: Protein ladder. Lane C control: uninduced culture. Lane 1–8: The culture incubation time: 1–8 h. A. The bacterial culture induced with 0.5 mM IPTG incubated at 37, (**e**) Purified Protein. Lane M-Protein marker, Lane 2-Control, lane3–4 Purified protein in buffer 5 with 200 mM and 500 mM imidazole concentration in elution buffer, respectively, (**f**) Zymography analysis of purified protein with 1% gelatin as substrate.

To screen for the bacteria producing the largest amount of protease, a skim milk assay was performed and among all isolates *Bacillus cereus* showed maximal activity (Fig. 1a). The bacteria were identified using 16S rRNA sequencing and BlastN analysis. The similarity was found to be 100% with the *Bacillus cereus* strain isolated from hydrothermal vent crabs. A phylogenetic tree was constructed using MEGA X software showing that *B. cereus* was closely related to *Bacillus thuringiensis* (Fig. 1b). The Serine protease gene from *B. cereus* was amplified and found to be approximately 1050 bps in size (Fig. 1c). The sequence analysis using BLASTn confirmed the amplified gene as alkaline protease with a similarity of 98.35%. The gene for this protease was then successfully cloned into a T & A cloning vector and further cloned into pET-32b (+) expression vector and transformed into *E. coli* BL21 [ECOS 101 (DE3)] expression cells. The colonies were confirmed by gene amplification by colony PCR and plasmid sequencing. Previously, the protease gene from *Bacillus* sp. was amplified and the size of the amplified product was 1100 bps. Further confirmation of deducing the amino acid sequence and activity was not reported^17^. Several other studies proved that *Bacillus* strains are optimal targets to study protease enzyme activity since they are known to produce the highest yields of proteases^28–31^.

### Purification, molecular mass determination, and mass spectrometry analysis

The culture supernatant was induced with different concentrations of IPTG and different temperature treatments were analyzed by SDS PAGE. The results showed the protein over expressed after 6 h of incubation. The optimal temperature for expression was 37 °C for 7 h after induction with 0.5 mM IPTG. At this concentration and time the desired protein expressed higher and the expression of other proteins was lower (Fig. 1d). The IPTG concentration of 1 mM resulted in the expression of non-targeted proteins (Supplementary Fig. S1a). The purification of proteins in different buffers showed phosphate buffer 20 mM at pH 7.5 and NaCl at 500 mM to be optimal for purification. Furthermore, the imidazole concentration of 200 mM in the solution buffer and 20 mM in the binding buffer resulted in purified soluble recombinant protein (Fig. 1e) whereas with other buffers and a higher concentration of imidazole resulted in non-specific protein elution (Supplementary Fig. S1b). From SDS PAGE the size of the protease was found to be 38 kDa. The protein was present as a monomeric single band. Gelatin (10 mg/mL) zymography showed the proteolytic activity of the protease enzyme (Fig. 1f) and a single band appeared after destaining for purified protein. The supernatant obtained from *Bacillus cereus* was also analyzed and two bands with different molecular weight were observed (Supplementary Fig. S1c).

**Figure 2:**
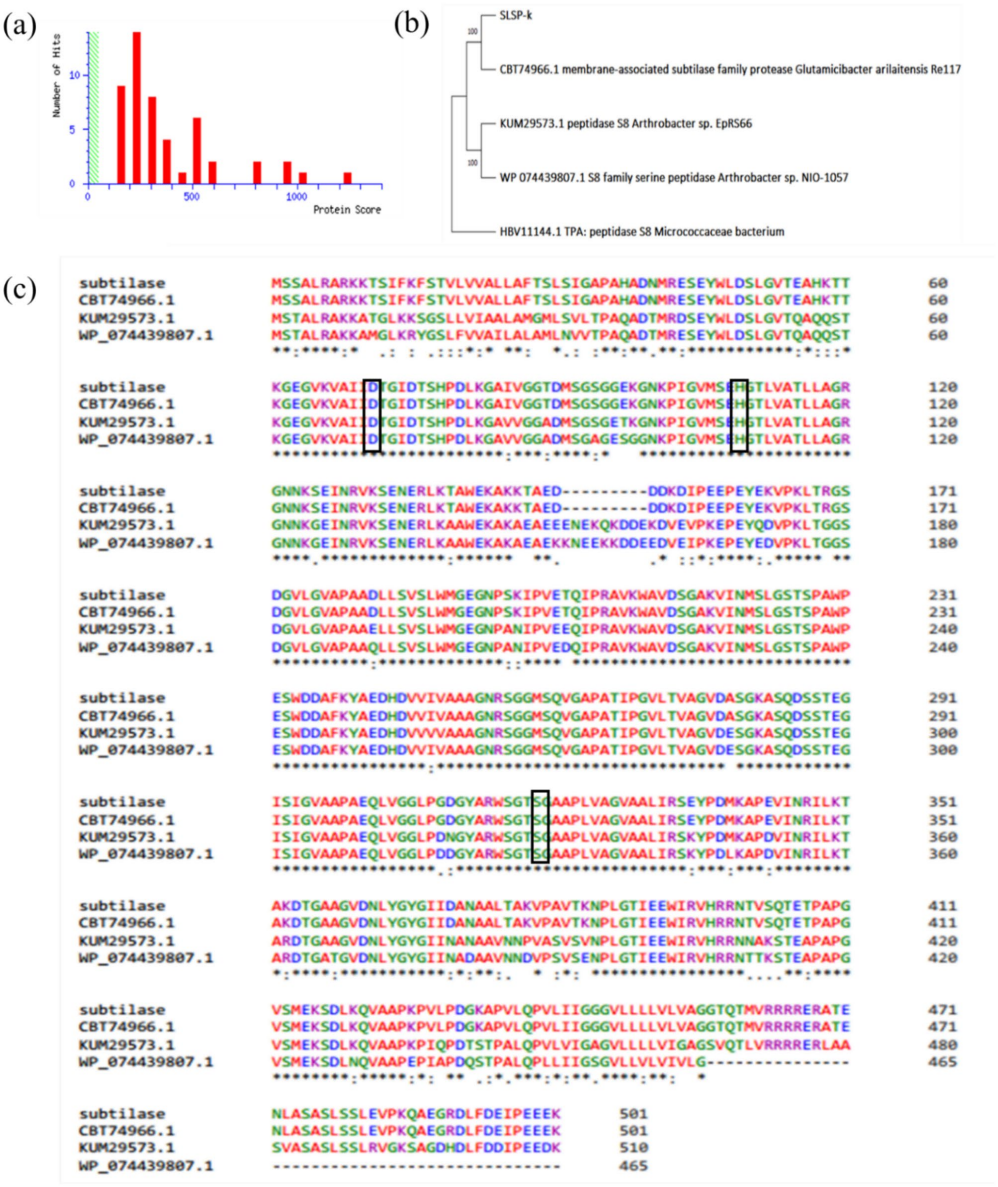
(**a**) Mascot Score Histogram, (**b**) Phylogenetic tree constructed using amino acid sequence of subtilisin like serine protease by Mega X software, (**c**) Homologous sequence alignment of protease using Clustal omega program. The catalytic triad is marked with black box.

The MASCOT score (Fig. 2a) from mass spectrometry analysis and the amino acid sequence retrieved were used in BLASTP search to find the protein similarity. The sequencing results predicted the protein as subtilisin-serine protease belonging to the MEROPS peptidase family S8. The results showed 99% similarity with the membrane-associated subtilase family protease from *Glutamicibacter arilaitensis* Re117 (accession number CBT74966.1). Similarity percentage with other species was only 44.07% which included the Enterobacteriaceae strains *Escherichia coli*, and *Klebsiella variicola.* A phylogenetic tree was constructed using the results of the BlastP analysis in MEGA-X software (Fig. 2b)*.* Homologous sequence alignment showed the sequence to be closely related to the serine protease S8 family (Fig. 2c). The sequence was similar to the protein CBT74966.1, KUM29573.1 and WP074439807.1. The conserved catalytic triad was observed at region 71-Aspartic acid, 109-Histidine, and 319-Serine. Structural similarity was found to be similar to subtilin protease and keratinase (see Supplementary Fig. S2). Purification and isolation of protease from *Bacillus* spp. was reported earlier by several researchers but there were no reports of cloning and characterization of subtilisin like serine protease with keratinolytic activity (SLSP-k) from *Bacillus cereus.* Previously reported data about serine proteases showed that the size of the protein varies between 20 and 60 kDa. Park et al.^32^ studied three alkaline serine proteases from the invertebrate polychaete *Cirriformia tentaculata* and their estimated molecular masses were found to be 28.8, 30.9, and 28.4 kDa^32^. Another study on a serine protease from the sea cucumber (*Stichopus japonicus*) was 34 kD^33^. The fish-derived myofibril-bound serine proteinase (MBSP) analyzed by sodium dodecyl sulfate–polyacrylamide gel electrophoresis (SDS- PAGE) showed a major protein band with a molecular weight of approximately 36 kDa^34^.

### Bioinformatic analysis

**Figure 3:**
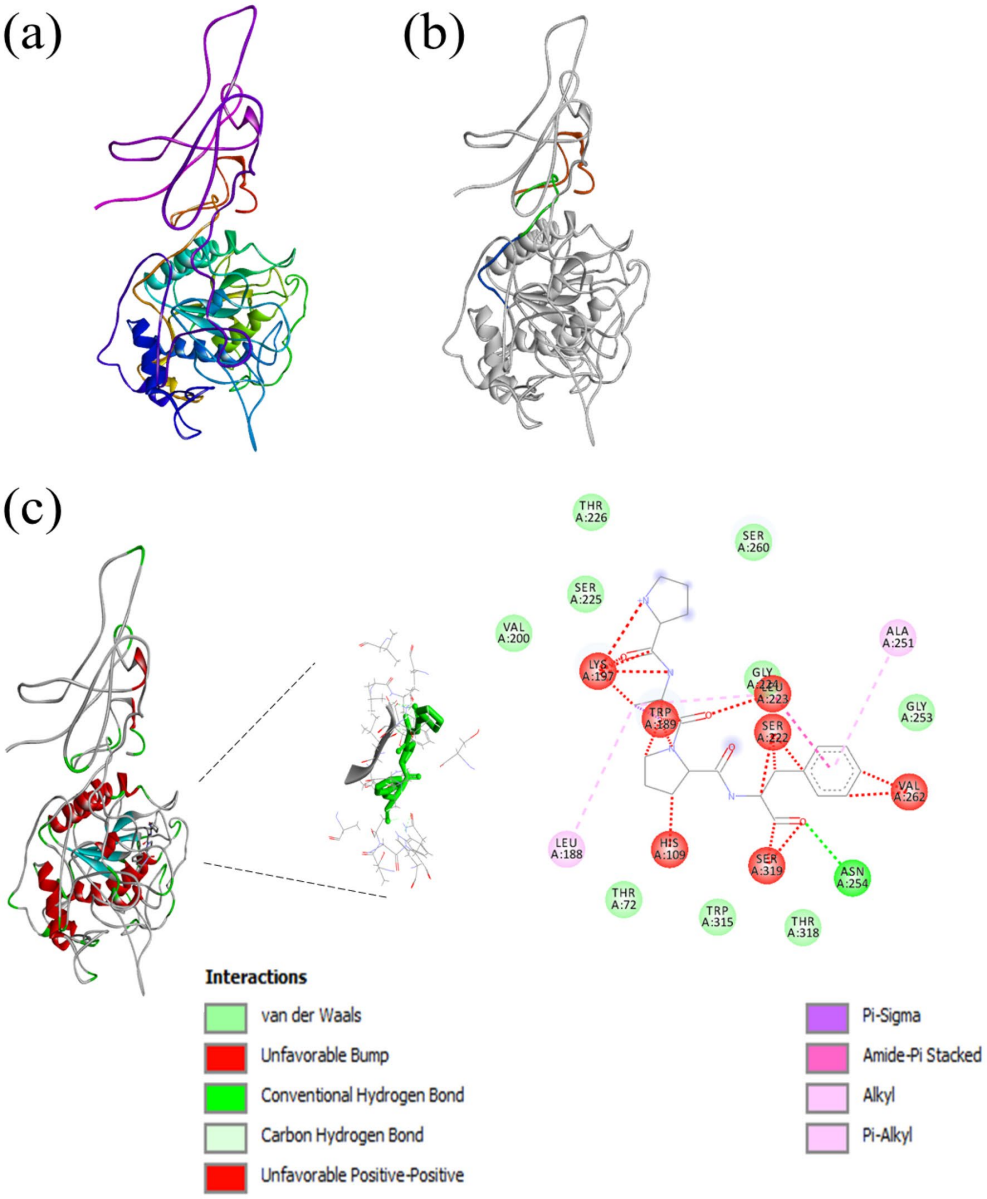
Structural predictions. (**a**) I-TASSER structure prediction (https://zhanglab.ccmb.med.umich.edu/I-TASSER/) and image retrieved from Discovery studio software (Discovery Studio Visualizer, v.17.2, San Diego: Dassault Systèmes, 2016), (**b**) The signal peptide region is colored, Red for N-terminal, Green for H-terminal and Blue for C-terminal (http://www.cbs.dtu.dk/services/SignalIP/), (**c**) The ligand binding site predicted by I-TASSER (109-HIS,189-TRP, 222-SER, 223-LEU, 224-GLY, 225-SER, 251-ALA, 253-GLY, 254-ASN, 318-THR, 319-SER) (https://zhanglab.ccmb.med.umich.edu/I-TASSER/).

The retrieved amino acid sequence was further analysed to detect the tertiary structure using SWISS-MODEL and I-TASSER^35^ (Fig. 3a). The C-Score of the predicted model was − 1.85 (commonly it is in the range of [− 5 to 2]). The signal peptide region as predicted by SignalP program showed the protein is extracellular and has an N-region of amino acids from 1 to 18, an H-region from 19 to 30, and a C-region from 31 to 38 (Fig. 3b). The possible ligand binding sites predicted by the I-TASSER tool are at positions 109-HIS, 189-TRP, 222-SER, 223-LEU, 224-GLY, 225-SER, 251-ALA, 253-GLY, 254-ASN, 318-THR and 319-SER (Fig. 3c).

### FTIR analysis of hydrolysed casein

**Figure 4:**
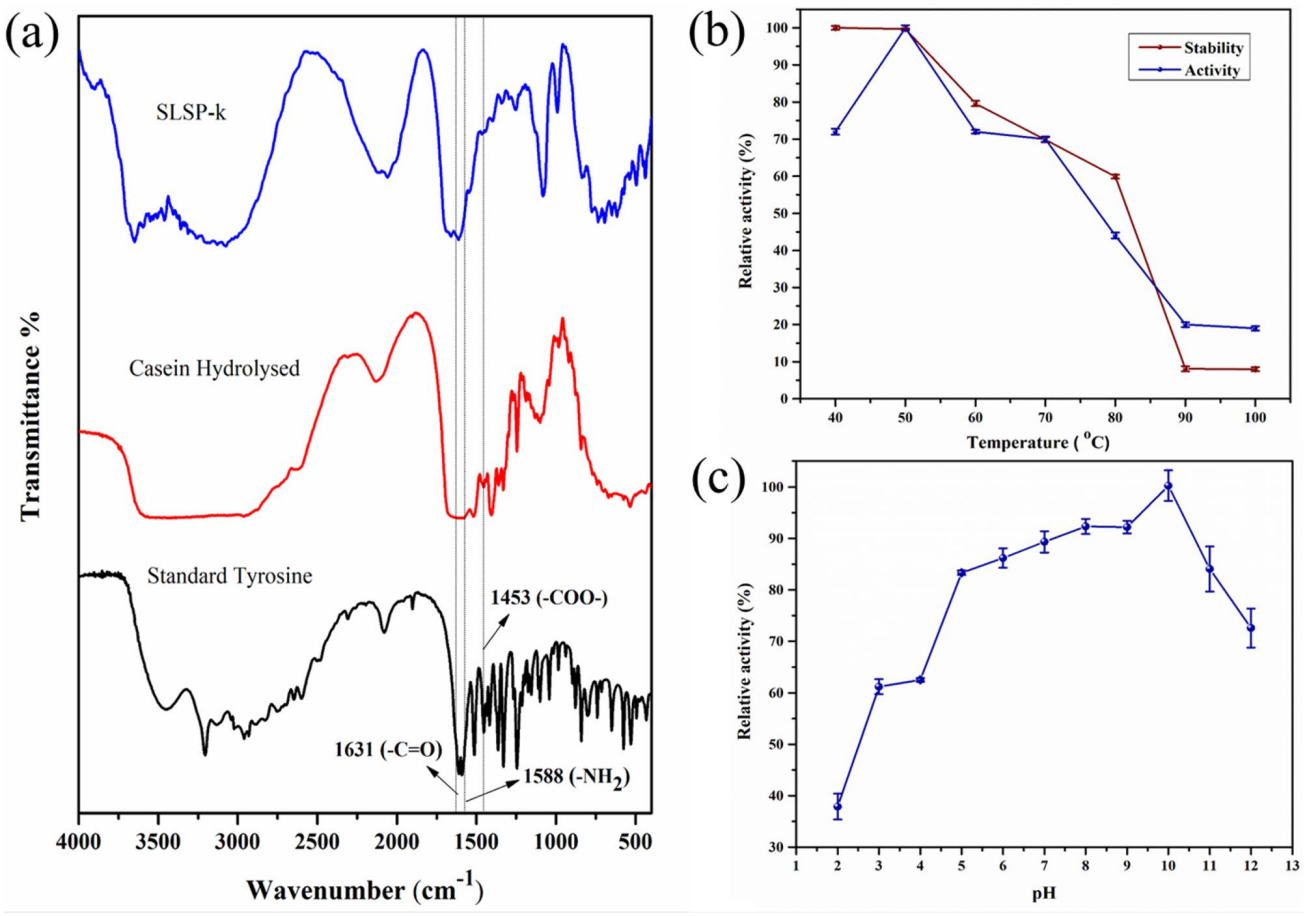
(**a**) FT-IR spectra of SLSP-k with casein as substrate, (**b**) Effect of temperature on activity and stability of SLSP-k, (**c**) Effect of pH on activity.

SLSP-k protease activity was predicted by using 0.6% casein as substrate. The spectra were similar to the standard l-tyrosine spectra and the spectra of hydrolyzed casein (see Fig. 4a). The peaks at 1465, 1602 and 1743 cm^−1^ corresponded to the stretching modes of the –COO–, –NH_**2,**_ and –C=O group, respectively^36^. The results obtained are in accordance with the results shown by Lakshmi et al. for purified alkaline proteases. The peaks obtained in their study are similar to the peaks obtained in our study^25^.

### Biochemical characterization of SLSP-k protease activity

#### Optimal temperature and stability

The optimal temperature of protease activity was at 50 °C with casein as a substrate (Fig. 4b). At temperatures beyond 50 °C, enzyme activity declined significantly. Thermal stability of subtilisin protein was up to 80 °C. However, at 80 °C the enzyme lost already 55% activity from the initial activity and at higher temperature the enzyme became inactive. The protein showed stability between 40 and 50 °C; with gradual temperature increase the stability decreased. At 80 °C the stability was reduced to 60% from its initial stability. At 90 °C the protein completely lost its stability due to denaturation. The observed data suggest that the protein is stable and can withstand a temperature up to 80 °C. Similar to our findings was shown for the alkaline serine protease from the pancreas of the hydrothermal vent inhabiting Gazami crab (*Portunus trituberculatus*) with optimal activity at 50 °C^37^. An alkaline protease belonging to subtilisin-like serine proteases produced by an endophytic *Bacillus halotolerans* exhibited an optimal activity at 50 °C^38^. In our study the bacterium *Bacillus cereus* was isolated from shallow hydrothermal vents where the temperature varied from 30 to 116 °C^1^. Such an environment certainly contributed to the high temperature stability of the protease SLSP-k (see Fig. 4b).

#### Optimal pH for protease activity

The optimal pH for SLSP-k was found to be pH 10 (Fig. 4c), though the protein was stable from neutral to alkaline pH. At neutral pH the activity was reduced by 20% when compared to pH 11. According to previous studies the subtilin like protease was mostly active at alkaline pH. For example, rBLAP is an alkaline serine protease retaining 80% activity at pH 8.0 with optimal activity at pH 12.8^39^. Another report claiming the same is the work done by Haddar et al. who isolated alkaline serine proteases from *Bacillus mojavensis* from marine water samples showing relative activities of about 80% and 71.7% at pH 11.0 and 12.0, respectively, compared to those obtained at pH 8.5^25^.

#### Effect of metal ions on subtilisin protease activity

The hydrolytic activity of SLSP-k in our study increased in the presence of the trace metals Ca^2+^, Co^2+^, Li^2+^, Mg^2+^, Mn^2+^, and Zn^2+^. Optimal activity was observed with 5 mM Mn^2+^. In the presence of 1 mM Mn^2+^ and 5 mM Co^2+^ it showed similar enhanced activity. Metals like Mg^2+^, Ca^2+^, Li^2+^, Zn^2+^, Co^2+^ showed similar effects on the activity of the protease at lower concentrations (1 mM) (Table 2). Enhanced activity in the presence of metals is probably due to the extremophilic hydrothermal vent site where the environment is enriched with heavy metals compared to the ambient environment^40^. The study also proved that the protein is stable and active in the presence of lower concentration (1 mM) of Hg^2+^. Metals like Cd^2+^, Cu^2+^ and Fe^3+^ completely inactivated protease activity at 5 mM concentration, whereas the activity decreased by 70% at 1 mM concentrations. There are similar reports where alkaline protease activity was significantly activated by Co^2+^ and Mn^2+^ and inhibited in the presence of Fe^2+^. Authors claimed that the presence of EDTA did not affect the protease activity^41^, whereas in our studies the activity was inhibited. Most of the findings on serine proteases suggest that Ca^2+^ enhanced the activity^37^. Thermotolerant alkaline serine protease from a novel species *Bacillus caseinilyticus* showed enhanced activity in the presence of Mg^2+^ and Ca^2+^^42^. Similarly, the activity of serine protease from *Geobacillus toebii* strain LBT 77 was stimulated by Ca^2+^ and Mg^2+^^43^. Joshi and Satyanarayana observed enhanced activity with Co^2+^ on rBLAP protease from *Bacillus lehesis* while Hg^2+^ reduced the activity of rBLAP^39^. Since the activity was enhanced in the presence of metal ions and also EDTA inhibited the activity, the protein can be classified as serine metalloprotease. The difference in the activity in the presence of metal ions with proteins is dependent on the concentration of each metal ion in cells and organelles which is within a specific range depending on the cells^44^. As mentioned by Smith (2015), many proteins are highly selective, limiting a binding motif to only one specific metal ion. Thus, proteins have evolved binding sites with specific spatial restrictions on the binding site preventing other metal ions from binding^45^. Depending upon bioavailability and nature along with climatic factors heavy metals may pose toxic or beneficial effects^46^. Heavy metals like Ni, Mn, and Zn are required for physiological processes in microbes, enzyme activity and stabilization of molecules^47^. Other heavy metals, like lead (Pb), arsenic (As), mercury (Hg), silver (Ag), cadmium (Cd), and gold (Au), are not required for body functioning^47^. The results reported by Lorenz (2006) showed that high levels of Cd and Cu significantly reduces the activity of protease, urease, alkaline phosphatase and arylsulphatase^48^. The reported reduced enzyme activity may be due to binding of Cd^2+^ to sulphydryl groups^49^.

**Table 2:** Residual activity of SLSP-k with different concentrations of metal ions.

Metal ions	Residual activity (%)	
	1 mM	5 mM
Ca^2+^	101.98 ± 1.90	59.96 ± 0.10
Co^2+^	94.81 ± 0.90	84.88 ± 0.90
Hg^2+^	64.25 ± 1.10	17.18 ± 1.10
Li^2+^	84.99 ± 1.19	76.82 ± 1.11
Mg^2+^	99.27 ± 1.90	65.27 ± 1.10
Mn^2+^	110.00 ± 1.09	122.00 ± 1.00
Ni^2+^	29.92 ± 0.10	23.51 ± 0.89
Zn^2+^	101.87 ± 0.90	68.48 ± 1.00
Cd^2+^	20.92 ± 1.70	14.18 ± 1.70
Cu^2+^	21.20 ± 1.10	2.57 ± 1.99
Fe^3+^	20.92 ± 1.90	1.88 ± 2.10
Control	100	100

#### Effect of inhibitors and surfactants on subtilisin like serine protease activity

Inhibitors are protein-specific and can be used for protein classification and activity studies. The activity was studied in the presence of inhibitors mentioned in Table 3. The protein was completely inhibited in the presence of PMSF even at a concentration of 1 mM. Since subtilisin belongs to the serine proteases, the activity should be inhibited in the presence of PMSF—which was actually observed in our study. EDTA had similar effects on protein-like PMSF, causing the loss of its activity. EDTA is a metal chelating agent and in its presence enzyme activity was inhibited by 70% in our study. This provides another proof of the necessity of metals for hydrolytic activity and stability. These findings are similar to earlier reports claiming that the structure of the protease from *B. licheniformis* had two Ca^2+^ binding sites and its removal caused a significant reduction of thermal stability and activity^50,51^. The alkaline protease studied by Thakur et al. was inhibited by EDTA (5 mM). This suggested it to be a metalloprotein^52^.

**Table 3:** Residual activity of SLSP-k in the presence of inhibitors and surfactants.

	Residual activity (%)	
	1 mM	5 mM
**Inhibitors**		
Control	100	100
PMSF	2.72 ± 0.80	2.62 ± 1.09
EDTA	2.72 ± 0.89	2.62 ± 0.90
DTT	88.19 ± 1.89	102.90 ± 1.24
**Surfactants**		
Control	100	100
Tween-20	95.54 ± 1.59	88.67 ± 0.89
Triton-X 100	94.04 ± 0.80	80.08 ± 1.09
SDS	68.62 ± 0.99	65.36 ± 1.98

The protein SLSP-k in our study was stable and showed 88% activity in the presence of 1 mM DTT, whereas at higher concentrations 102% activity was observed (Table 3). Treatments of surfactants showed that the protein SLSP-k was stable and showed hydrolysis when treated with Triton X 100, Tween 20, and also with strong detergents like SDS (Table 3). The protein was 90% active when treated with 0.5% of Tween 20 and 1% of Triton X 100. The stability was 70% when treated with 0.5% and 1% SDS. The results demonstrated that this enzyme can withstand and shows proteolytic activity in the presence of surfactants like Tween 20 and Triton X-100 and surfactants like SDS at 0.5 and 1% concentration. Therefore, this protein can be used in several commercial applications, such as for the production of detergents.

#### Organic solvent effects

Organic solvent effects on protein stability were found to be almost similar. The solvent acetonitrile provided a maximal stability of 65% to SLSP-k protease, followed by methanol, ethanol and DMSO, all providing 55% stability. The lowest stability of 53% was observed in ethyl acetate (Table 4). Since the protein did not lose its activity we can say that the protein is stable enough to hydrolyse casein. However, NaCl though reduced the activity by 30% but stability was still observed. The work by Thakur et al. demonstrated maximum stability of the protease in methanol and minimum stability in iso-amyl alcohol. The proteases purified by Thakur et al. showed stability in the presence of detergents but there was decreased enzyme activity in the presence of NaCl^52^. Enzyme reactions in organic solvents are of increasing industrial interest specially with protein having greater solubility in organic solvents, e.g. during the biosynthesis of peptides.

**Table 4:** Residual activity of SLSP-k in the presence of solvents.

Solvent	Residual activity (%)
Control	100
2-Propanol	54.74 ± 2.09
Acetone	54.87 ± 1.90
DMSO	58.02 ± 0.90
Ethanol	57.98 ± 0.97
Ethyl acetate	53.28 ± 1.90
Methanol	58.03 ± 0.90
NaCl	69.09 ± 1.90

### Kinetic studies of protease SLSP-k

The K_m_ and V_max_ value calculated for protease SLSP-k using different concentrations of casein as a substrate at 50 ℃, pH 10 was 0.64 mM and 420 µmol/mL min, respectively (Supplementary Fig. S3). This was further estimated by applying the Lineweaver–Burk plot. The Km and Vmax values of the serine protease studied by Alici and Arabaci was 0.4 mM and 3333.3 μmol tyrosine/mL min, respectively^53^. In another study on extracellular alkaline proteases the K_m_ and V_max_ value of the purified protease using casein as substrate was 7.0 mg/mL, 54.30 µmol/min, respectively^54^.

### Keratinolytic activity: degradation of feather and human hair

**Figure 5:**
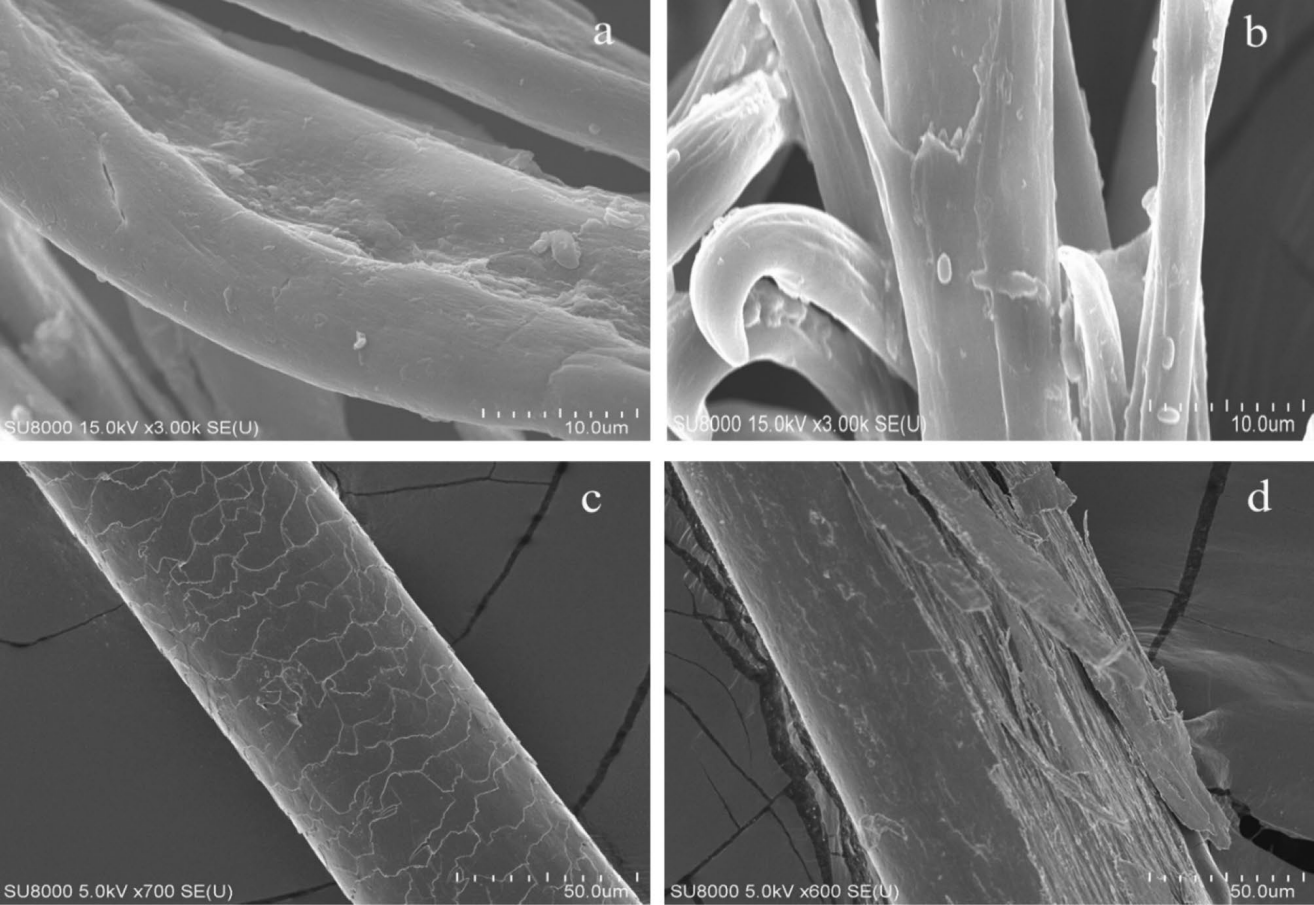
SEM images. (**a**) Control chicken feather, (**b**) Degraded chicken feather by treatment with SLSP-k incubated for 48 h at 50 °C, (**c**) Control human hair, (**d**) Degraded human hair by SLSP-k incubated for 72 h at 50 °C.

Gene sequencing and structural similarity showed that the protein gene sequence had a similarity with the keratinase gene (Supplementary Fig. S4). Since keratinases belong to serine proteases, their activity is inhibited by PMSF. The same was confirmed by our results. Also, keratinases are highly stable at a wide range of temperature and pH, and had a high affinity for metals. Our study found the same for the SLSP-k protease. To confirm the keratinase activity of degrading keratin in chicken feather and human hair, these items were treated with SLSP-k protease. Our SEM results proved that the enzyme was capable of degrading feather in 48 h at 50 °C with untreated feather as control (Fig. 5a,b) and human hair in 72 h at 50 °C with non-treated samples as a control (Fig. 5c,d). The keratin layer was completely degraded by SLSP-k protease. Another protease with keratinase activity studied in the literature was the recombinant MtaKer (rMtaKer) protease cloned from *Meiothermus taiwanensis* WR-220 belonging to the group of Terrabacteria, collected from Wu-rai Hot Spring located in northern Taiwan. This protease was classified as keratinase which showed similarity with subtilisin serine proteases. Keratinolytic activity was studied at 65 °C for 48 h and highest activity was found at pH 10 and 65 °C^55^. In another report an extracellular keratinase (KERUS) with a molecular mass of 29,121.11 Da was isolated from *Brevibacillus brevis* strain US575 with an optimal activity observed at 40 °C and pH 8. The keratinolytic activity on feather-degradation proved it as an alternative source for waste management and the production of value-added products^56^. Nnolim and Nwodo optimized chicken feather formulations for the optimum production of keratinase by *Bacillus* sp. CSK2. The keratinase exhibited maximum catalytic activity at pH 8.0 and could withstand a temperature range of 60–80 °C^57^. Another study on the strain *Bacillus pumilus* produced keratinase which could hydrolyse both alpha-and beta keratin. The protein sequence alignment indicated that this protease belonged to the S8 family which is a subtilisin like serine protease, similar to our protease with the molecular weight of 38 kDa^58^. Moridshahi et al. isolated a keratinase from *Bacillus zhangzhouensis* with a molecular weight of 42 kDa belonging to the serine proteases. This protease showed maximum activity at a temperature of 60 °C and a pH of 9.5. Similar to our studies the enzyme was stable in solvents like acetone, methanol, ethanol, DMSO, and also showed stability in detergents like Triton X-100 and Tween-80. In the presence of DTT there was an increase in its hydrolase activity. This activity was also increased in the presence of metal ions Mn^2+^, Ca^2+^, Na^+^, and K^+^^59^. *Bacillus pumilus* isolated from poultry exhibited high feather degradation. As discovered by our study, this keratinase was classified as a serine protease. The keratinase activity was enhanced in the presence of Mg^2+^ and Ca^2+^^60^. An acidophilic *Bacillus* sp. Okoh-K1 was shown to optimally produce extracellular keratinase and showing remarkable stability in the presence of reducing agents, surfactants, organic solvents, and laundry detergents^61^.

## Conclusion

A novel keratin degrading protease (SLSP-k) from a extremophilic shallow HV inhabiting bacterium with a molecular weight of 38 kDa was purified and characterized. Blastn analysis showed gene similarity with both serine protease and keratinase. Mass spectrometry analysis and structure analog prediction confirmed that the protein belongs to the subtilisin family of peptidases and has a similarity with keratinases. Hydrolysis activity was confirmed with casein as a substrate and keratinase activity with feather and human hair degradation as observed by SEM. The novel SLSP-k protease is stable at a wide range of temperature, pH, solvents, and detergents. This protein has, therefore, potential applications in commercial product making such as the production of detergents and in peptide synthesis research. Keratinase activity of the protein makes it suitable for applications in animal waste treatment like feather or hair degradation, for the production of animal feed, and in the leather industry for dehairing.

## Supplementary Information

Supplementary Information.
