# Supplementary material for: Novel recombinant keratin degrading subtilisin like serine alkaline protease from Bacillus cereus isolated from marine hydrothermal vent crabs
Source: Sci Rep. 2021 Jun 7;11:12007. doi: 10.1038/s41598-021-90375-4 (PMC8185006; doi:10.1038/s41598-021-90375-4)
Supplement: Supplementary file 1 — Supplementary Information. [file 41598_2021_90375_MOESM1_ESM.docx]

**<Scientific Reports>**

**Novel recombinant keratin degrading subtilisin like serine alkaline protease from *Bacillus cereus* isolated from marine hydrothermal vent crabs**

Revathi Gurunathan^1,2^,  [Bin Huang](https://pubmed.ncbi.nlm.nih.gov/?term=Huang+B&cauthor_id=29112129)^2,3^, Vinoth Kumar Ponnusamy^1,7^, Jiang-Shiou Hwang^4,5,6**^, Hans-Uwe Dahms^2,7,8*^

**^1^** Department of Medicinal and Applied Chemistry, Kaohsiung Medical University, Kaohsiung City-807, Taiwan

**^2^** Department of Biomedical Science and Environmental Biology, Kaohsiung Medical University, Kaohsiung City-807, Taiwan

**^3^** Regenerative Medicine and Cell Therapy Research Center, Kaohsiung Medical University, Kaohsiung 80708, Taiwan

**^4^** Institute of Marine Biology, National Taiwan Ocean University, Keelung 20224, Taiwan.

**^5^** Center of Excellence for Ocean Engineering, National Taiwan Ocean University, Keelung 20224, Taiwan.

**^6^** Center of Excellence for the Oceans, National Taiwan Ocean University, Keelung 20224, Taiwan.

**^7^** Research Center for Environmental Medicine, Kaohsiung Medical University, Kaohsiung City-807, Taiwan

^8^ Department of Marine Biotechnology and Resources, National Sun Yat-Sen University, Kaohsiung City-804, Taiwan

* Corresponding Author’s e-mail ID: [hansd@kmu.edu.tw](mailto:hansd@kmu.edu.tw)

** Co-corresponding author: [jshwang@mail.ntou.edu.tw](mailto:jshwang@mail.ntou.edu.tw)

**(a)**


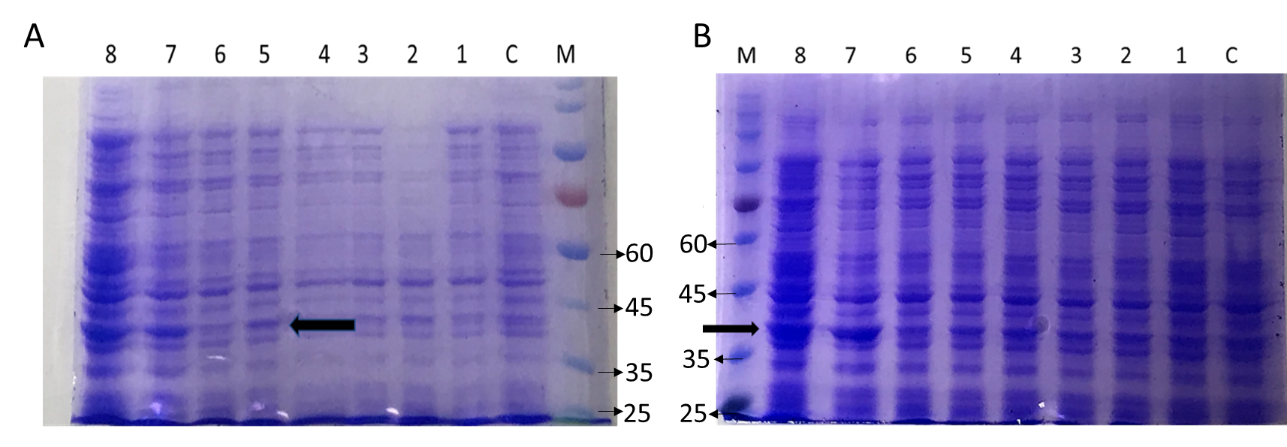


**(b)**


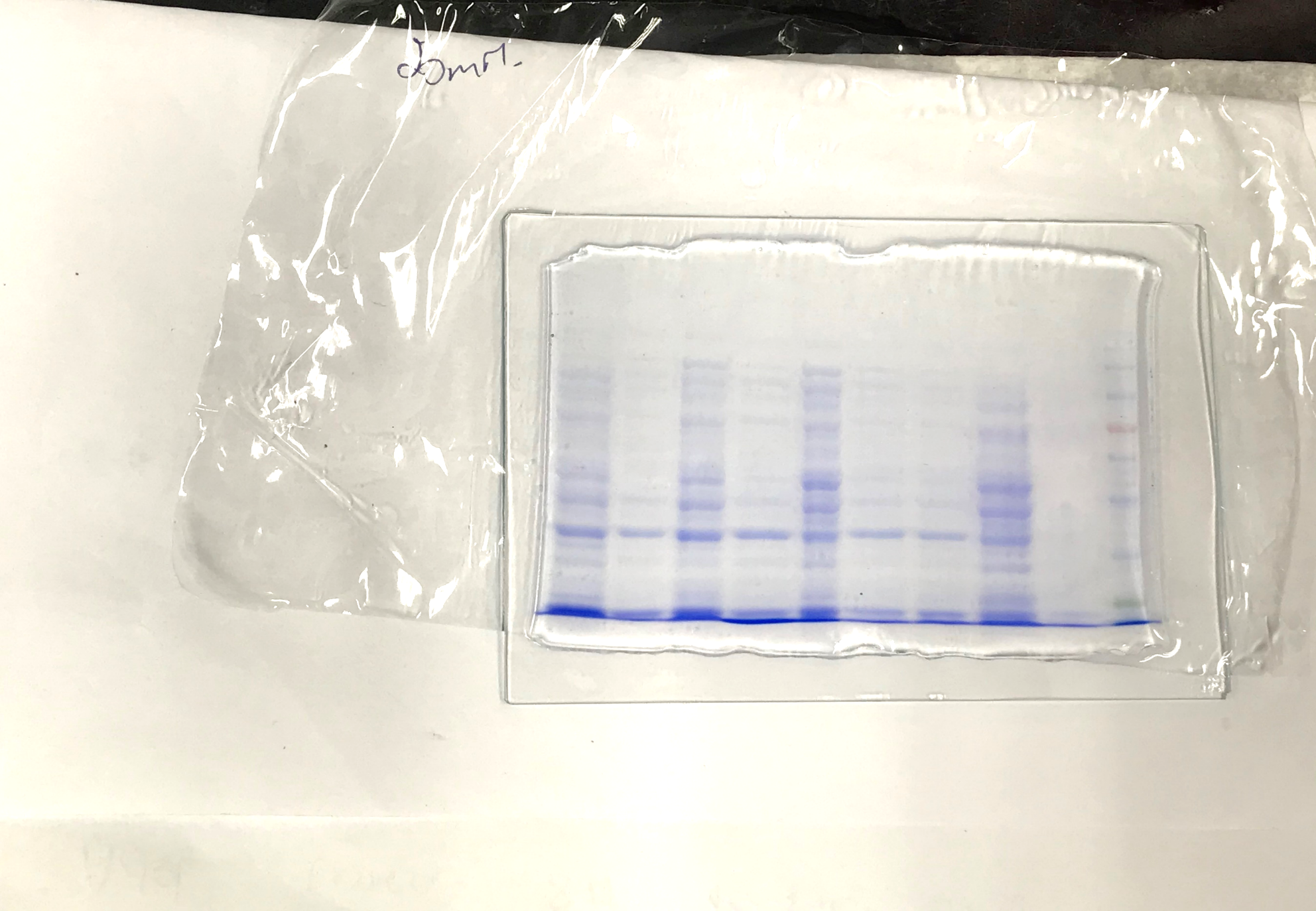


75

60

45

35

25

9 8 7 6 5 4 3 2 1 M

**(c)**


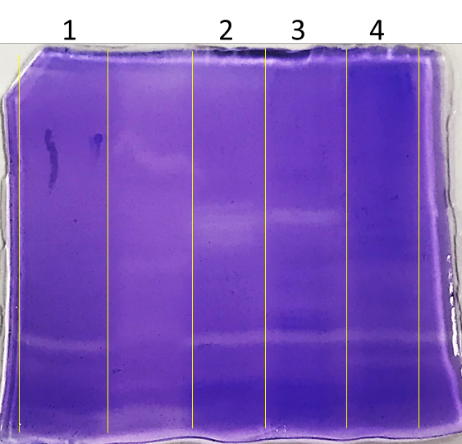


**Fig. S1. (a)** The SDS-PAGE analysis of protein expression. Lane M: Protein ladder. Lane C control: uninduced culture. Lane 1-8: The culture incubation time: 1-8hrs. A. The culture induced with 1mM IPTG was incubated at 37 ℃. **(b)** SDS-PAGE analysis of purified protein. Lane M - Protein marker, Lane 2 - Control, lane 3-4 purified protein in buffer 5 with 200 mM and 500 mM imidazole concentration in elution buffer, respectively. Lane 5 control, Lane 6 purified protein in buffer 3 with 500 mM imidazole concentration in elution buffer. Lane 7 control, Lane 8 purified protein in buffer 4 with 200 mM imidazole concentration in elution buffer, Lane 9 purified protein in buffer 1 with 200 mM imidazole concentration in elution buffer. **(c)** Zymography analysis of purified protein with 1 % gelatin as substrate with 200 mM imidazole. Lane 1- Purified protein, Lane 2-3 *Bacillus cereus* culture supernatant, Lane 4 - Non-purified protein.


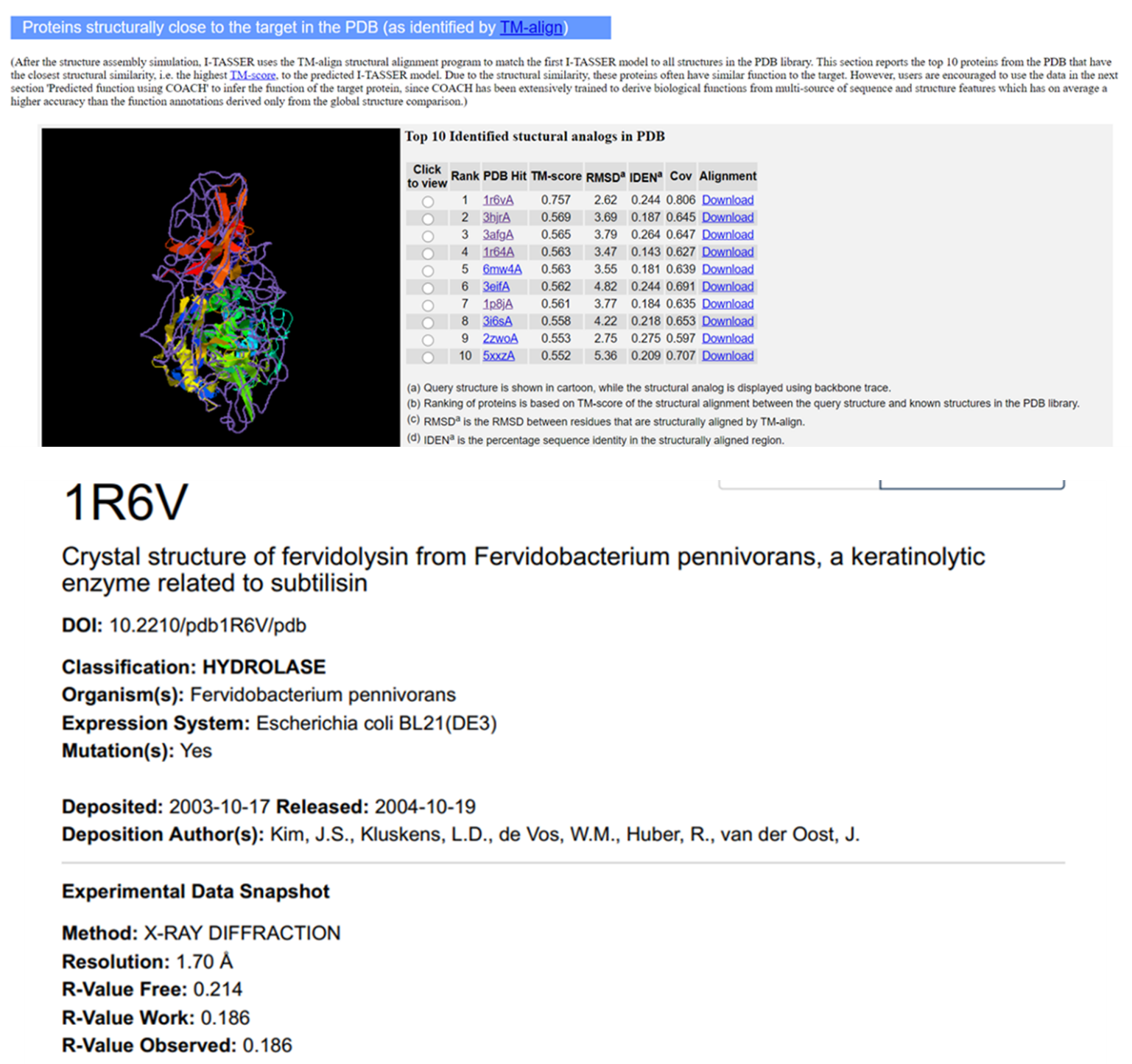


**Fig. S2.** The structural analog of SLSP-k.

**Fig. S3.** Lineweaver–Burk plot. The calculation of Km and Vmax value. 0.64mM and 420 umol/mL, respectively.


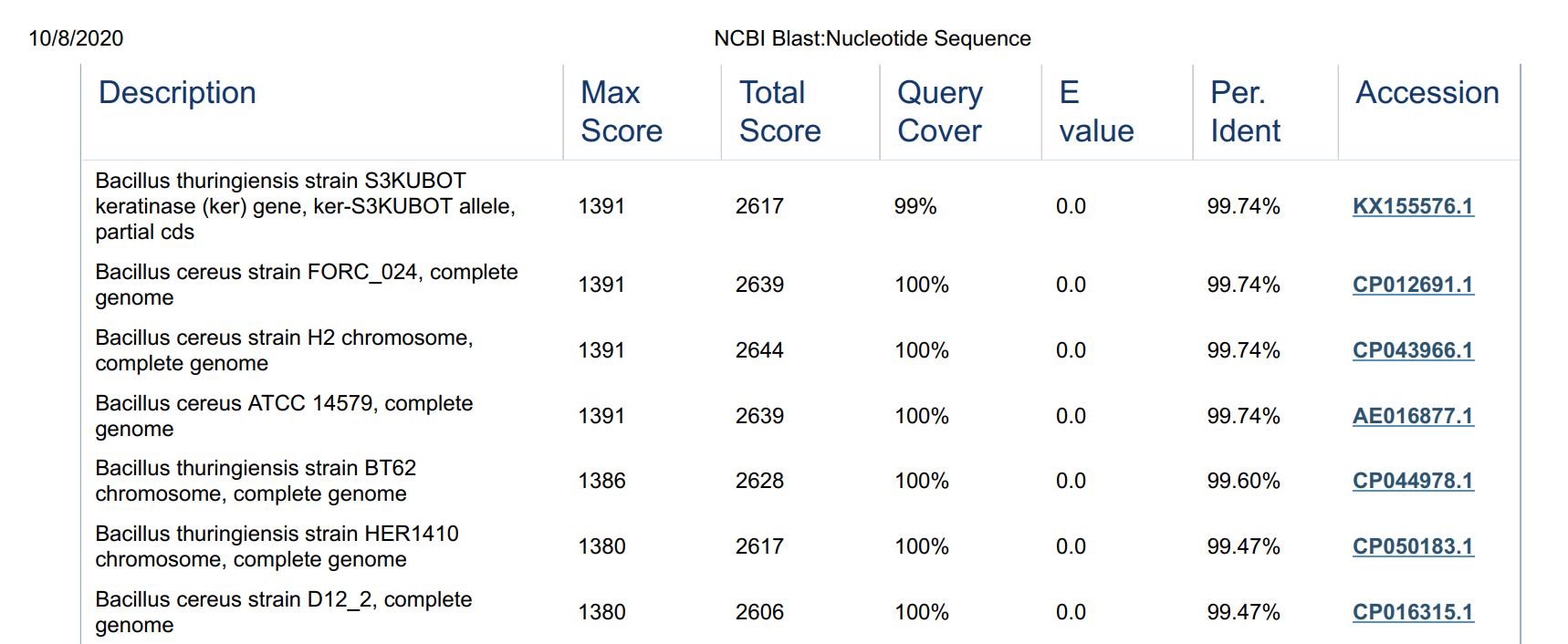


**Fig. S4.** NCBI Blast results of SLSP-k gene showing similarity with keratinase gene.
